# Antimicrobial resistance surveillance in Africa: Successes, gaps and a roadmap for the future

**DOI:** 10.4102/ajlm.v7i2.924

**Published:** 2018-12-06

**Authors:** Samuel Kariuki, Karen H. Keddy, Martin Antonio, Iruka N. Okeke

**Affiliations:** 1 Kenya Medical Research Institute, Nairobi, Kenya; 2 Faculty of Health Sciences, University of the Witwatersrand, Johannesburg, South Africa; 3 World Health Organization Collaborating Centre for New Vaccines Surveillance, Medical Research Council Unit, at London School of Hygiene & Tropical Medicine, Banjul, The Gambia; 4 Division of Microbiology & Immunity, Warwick Medical School, University of Warwick, Warwick, United Kingdom; 5 Department of Pharmaceutical Microbiology, University of Ibadan, Ibadan, Nigeria

Since the World Health Organization (WHO) launched the Global Antimicrobial Resistance Surveillance System (GLASS) in 2015,^1^ there has been rapidly growing awareness among many African countries that they need to be doing more to combat antimicrobial resistance (AMR). The Africa Centres for Disease Control and Prevention (CDC) was officially inaugurated in January 2017^2^ and will support countries commencing surveillance for serious infectious disease threats in Africa, including resistance. Review of the recent WHO GLASS report suggests that, while certain nations do have some surveillance systems in place, very few countries in Africa currently conduct effective routine surveillance.^1^ As editors of this special edition, our experience confirms this observation. Nonetheless, we were encouraged by the range and scope of articles we received when the call for this special edition went out. The subjects covered include multi-drug resistant tuberculosis,^3,4,5^ which remains a critical public health threat on the African continent, nosocomial infections,^6,7^ including those due to *Clostridium difficile*^8^, laboratory methods and technical issues,^9,10^ including external quality assessment^11^ and molecular methods,^3,12,13^ and malaria.^14^ Community-acquired resistance was underrepresented, although there was a major review on AMR in *Vibrio cholerae* O1.^15^ The articles included opinion pieces, general and systematic reviews and original research. What we missed was more original research with a wider range of country submissions and better regional representation, more articles looking at One Health, veterinary medicine and AMR, more studies with data on AMR in community-acquired pathogens and AMR trend data by country, including bacteria, fungi and parasitic infections, and better detail on antiretroviral resistance in HIV infection. We are pleased to find that the special issue’s Call for Papers increased the overall frequency of submissions in this area and so subsequent regular issues of the journal are likely to continue the exposition of AMR surveillance in Africa that this issue has initiated.

Omissions notwithstanding, what is represented in this issue is a great and promising start: GLASS and the Africa CDC are both still in their infancy and no country can be expected to begin with comprehensive surveillance. What we have seen and present in this issue is an architect’s rough sketch of where we can go as a continent. Readers should understand that surveillance for AMR, irrespective of the pathogen or the setting, is not about the academic exercise of data collection but is a practical and powerful tool that is critical in defining optimal interventions both in local health settings and for what has become a global health crisis.

When AMR surveillance data are published in a journal, compared with submitting national data to a supra-governmental organisation, these data need not be fully comprehensive or subscribe to a predetermined list of pathogens or antimicrobials. The articles in this special edition hence provide a valuable snapshot of what can be done in Africa, rather than a comprehensive analysis of the AMR surveillance in Africa. Readers of this special edition, which emphasised the need for planning and progress as well as AMR data, should be able to see beyond the AMR data published here to what is being achieved by their continental neighbours, recognising that they too have the potential to do similar work. Remembering that imitation is the highest form of flattery, we can use the experience of our neighbours, start small and grow our surveillance systems. There are new resources that we can access through the WHO^1^ and Africa CDC^2,16^ to support us. The responsibility rests with us to find the will and the way to take surveillance for AMR to the next phase of effective implementation.

We all concede that there are numerous challenges we face in our attempt to implement effective and sustainable AMR surveillance programmes in Africa. These challenges range from inadequate resources and weak supply chains for consumables for microbiological laboratory procedures, lack of human resources well trained in AMR surveillance and poor or lack of commitment by facility management to embrace AMR as a healthcare issue. Where facilities do exist, under-utilisation is common. However, despite the lack of infrastructure and other resources required to perform optimal surveillance for AMR, most laboratories in Africa can still generate, collate and disseminate quality data, albeit with some ingenuity. If there is a lack of samples for systematic evaluation of AMR trends, mutually beneficial partnerships between centres responsible for coordinating surveillance and local hospitals or healthcare facilities can jumpstart focused surveillance on specific organisms. Such centres can then gradually expand the surveillance to include more pathogens and more facilities. Indeed, we could perform systematic sentinel surveillance so as to generate reliable, accurate and quality-controlled data, using the minimal resources typically available in our settings, for implementation of infection control and prevention programmes. Similarly, livestock-serving veterinary laboratories could target a component of the meat value chain and purposively sample within their capacity, for example, one in every 10 meat swabs in the local abattoir for AMR testing and surveillance. This effort can additionally be embedded in the public health requirement for ensuring food safety. Quality routine culture and susceptibility testing can easily and cost-effectively provide high-quality surveillance data. However, as Omar et al.,^3^ Smith,^12^ Gellband^13^ and Mohammed et al.^15^ all demonstrate, additional valuable information can be gleaned from surveillance systems that use whole genome sequencing and this is something that developing systems should consider. For quality assurance, which is a hallmark of a standard surveillance system, it will be important for laboratories to be registered and participate in an external quality assessment scheme operating to internationally recognised standards, some of which are supported by WHO and other partners and are therefore potentially more affordable for laboratories in our African setting.

Implementation of national action plans for AMR surveillance from a One Health approach will be critical in ensuring sustainability of the collaborative, multi-sectoral, and trans-disciplinary efforts at the local, regional and national level. Leveraging resources that can be jointly deployed both in-country and across national borders for sample collection, laboratory analysis, and data capture, analysis and dissemination, will optimise the use of resources to achieve the desired goals, providing evidence for action to prevent and contain AMR. On funding AMR surveillance activities and for a sustainable AMR surveillance programme, it will be critical that budget commitments made by national governments to support the national action plan activities are adequately maintained. This too will require some ingenuity so as to demonstrate that data from AMR surveillance actually impact on delivery of healthcare, food security and sustenance of a healthy environment. Thus, governments are constantly reminded that good surveillance systems are actually cost efficient, particularly in the long term. We need to work with health economists to deliver data on those economic costs associated with a lack of action on the AMR menace in our countries in a language that policymakers will easily understand. In the meantime, we will need to utilise the resources currently available through various initiatives, such as the tripartite United Nations agencies (WHO, the Food and Agriculture Organization and the World Organization for Animal Health), as well as other partners who have specific interests in promoting and supporting One Health AMR surveillance in Africa. The outlines of two successful routes to national surveillance, Ibrahim et al.^17^ and Opintan^18^, showcase how donor funds were leveraged to support local planning and initiatives in Ethiopia and Ghana. In making the most of external initiatives and opportunities, however, we must remember that these will only serve as short- and medium-term solutions – we have to own the process of fully implementing our national action plans, using our own resources, if AMR surveillance and containment will be sustainable, and serve our principal national goals and objectives.

## Sources of support

This special issue was supported by the Bill and Melinda Gates Foundation (OPP1178631) and by the Centre for Disease Dynamics, Economics and Policy.
